# Supplementary material for: Estimating the Direct Disability-Adjusted Life Years Associated With SARS-CoV-2 (COVID-19) in the Republic of Ireland: The First Full Year
Source: Int J Public Health. 2022 Jun 2;67:1604699. doi: 10.3389/ijph.2022.1604699 (PMC9200950; doi:10.3389/ijph.2022.1604699)
Supplement: Supplementary file 1 [file DataSheet1.docx]

**Estimating the direct Disability-Adjusted Life Years (DALYs) associated with SARS-CoV-2 (COVID-19) in the Republic of Ireland: The first full year**

**Appendix 1: Mortality Input Data (Cork, Ireland. 2021). (Page 2.)**

**Appendix 2: Morbidity Input Data (Cork, Ireland. 2021). (Page 3.)**

**Appendix 3: Years of Life Lost Calculation (Cork, Ireland. 2021). (Page 2.)**

**Appendix 4: Years Lived with Disability Calculation (Cork, Ireland. 2021). (Page 3.)**

**Appendix 5: Disability Adjusted Life Years Calculation (Cork, Ireland. 2021). (Page 3.)**

**Appendix 6: Sensitivity Analysis (“Post Acute Consequences” “Transition Probability” 26.6%, Duration 56 Days) (Cork, Ireland. 2021). (Page 6.)**

**Appendix 7: Overview of Published National Burden of COVID-19 studies, Published between January 2020 and December 2021. (Cork, Ireland. 2021). (Page 5.)**

| **Country** | **Period of analysis** | **Estimation of total symptomatic infected** | **Reference life expectancy table** | **Long -COVID included** | **DALY/100,000** | **% YLD** |
| --- | --- | --- | --- | --- | --- | --- |
| Australia ^a^ ^(28)^ | 01 Jan -31 Dec 2020 | No (notified positives only) | GBD-2010 | Yes, estimated | 32.7 | 3.5% |
| Germany | 01 Jan -31 Dec 2020 | No (notified positives only) | Germany 2016/2018 | No | 368 | 0.7% |
| Malta | 7 Mar 2020-31 Mar 2021 | Yes (notified positives adjusted for under ascertainment) | GBD-2019 | Yes, limited | 1086 | 5% |
| Scotland | 1 Jan-31 Dec 2020 | Yes (SEIR modeling) | GBD-2019 | Yes, limited | 1770- 1980 | 2% |

^a^ Australian Burden of Disease Study completed in cooperation with burden-eu’s task Force.
